# Safety and efficacy of percutaneous coronary intervention versus coronary artery bypass graft in patients with STEMI and unprotected left main stem disease: A systematic review & meta-analysis

**DOI:** 10.1016/j.ijcha.2022.101041

**Published:** 2022-04-25

**Authors:** Talal Almas, Ahson Afzal, Hameeda Fatima, Sadia Yaqoob, Furqan Ahmad Jarullah, Zaeem Ahmed Abbasi, Anoosh Farooqui, Duaa Jaffar, Atiya Batool, Shayan Ahmed, Neha Sara Azmat, Fatima Afzal, Sarah Zafar Khan, Kaneez Fatima

**Affiliations:** a Department of Medicine, Dow University of Health Sciences, Karachi, Pakistan; b Department of Medicine, Shaheed Mohtarma Benazir Bhutto Medical College, Karachi, Pakistan; c Department of Medicine, Jinnah Medical & Dental College, Karachi, Pakistan; d Department of Medicine, United Medical and Dental College, Karachi, Pakistan; e Department of Medicine, Karachi Medical and Dental College, Karachi, Pakistan; f Department of Medicine, RCSI University of Medicine and Health Sciences, Dublin, Ireland

**Keywords:** Percutaneous coronary intervention, Coronary artery bypass graft, Unprotected left main coronary artery disease, Meta-analysis

## Abstract

**Introduction:**

Owing to its large area of supply, left main coronary artery disease (LMCAD) has the highest mortality rate among coronary artery lesions, resulting in debate about its optimal revascularization technique. This *meta*-analysis compares percutaneous coronary intervention (PCI) versus coronary artery bypass grafting (CABG) for the treatment of LMCAD.

**Method:**

MEDLINE, TRIP, and Cochrane Central databases were queried from their inception until 25 April 2021, to determine MACCE (major adverse cardiac and cardiovascular events), all-cause mortality, repeat revascularization, myocardial infarction (MI) and stroke rates post-revascularization for different follow-ups. 7 RCTs and 50 observational studies having 56,701 patients were included. A random-effects model was used with effect sizes calculated as odds ratios (odds ratio, OR).

**Results:**

In the *short term (1 year)*, PCI had significantly higher repeat revascularizations (OR = 3.58, 95% CI 2.47–5.20; p < 0.00001), but lower strokes (OR = 0.55, 95% CI 0.38–0.81; p = 0.002). In the *intermediate term (2*–*5 years)*, PCI had significantly higher rates of repeat revascularizations (OR = 3.47, 95% CI 2.72–4.44; p < 0.00001) and MI (OR = 1.39, 95% CI 1.17–1.64; p = 0.0002), but significantly lower strokes (OR = 0.54, 95% CI 0.42–0.70; p < 0.0001). PCI also had significantly higher repeat revascularizations (OR = 2.58, 95% CI 1.89–3.52; p < 0.00001) in the *long term (≥5 years),* while in the *very long term (≥10 years),* PCI had significantly lower all-cause mortalities (OR = 0.77, 95% CI 0.61–0.96; p = 0.02).

**Conclusion:**

PCI was safer than CABG for patients with stroke for most follow-ups, while CABG was associated with lower repeat revascularizations. However, further research is required to determine PCI’s safety over CABG for reducing post-surgery MI.

## Highlights

• The advent of percutaneous coronary intervention (PCI) has caused much debate about the optimal revascularization method for LMCAD.
• Several studies have previously been conducted comparing PCI versus CABG for LMCAD, however most of these have only taken into account randomized controlled trials (RCTs), while ignoring observational studies.
• This study takes data from both RCTs and observational studies to obtain a better comparison of the two revascularization techniques.
• It is the first meta-analysis to report data for various adverse outcomes after 10 years of follow-up.

## 1 Introduction

Coronary artery disease is a major cause of morbidity and mortality in developed countries [1] Coronary artery disease involving stenosis of the left main artery, or left main coronary artery disease (LMCAD) has the highest mortality of any coronary lesions owing to its vast area of supply [2]. Significant LMCAD is defined as more than 50% angiographic narrowing of the artery and is found in about 4 to 6 % of the patients undergoing coronary angiography [3]. Because of its vital significance, the optimal revascularization technique for LMCAD has been a topic of much debate.

Coronary Artery Bypass Grafting (CABG) had been the main revascularization procedure for LMCAD for several decades, but with the advent of modern minimally invasive techniques, Percutaneous Coronary Intervention (PCI) has emerged as an acceptable alternative. The 2017 US appropriate use criteria and the 2018 European Guidelines suggest PCI as an appropriate alternative to CABG in patients with LMCAD and low-to-intermediate anatomical complexity. [4].

Our *meta*-analysis aims to compare the safety and efficacy of PCI versus CABG in treating LMCAD for different follow-up periods. Several studies have previously been conducted on this topic, however most of the previous *meta*-analyses comparing PCI versus CABG for LMCAD have only taken into account randomized controlled trials (RCTs), while ignoring observational studies. While RCTs are considered to be more reliable, observational studies are said to give a more accurate representation of “real world” data, therefore in this study, we are also pooling data from observational studies in addition to RCTs, to analyze the adverse outcomes such as MACCE (major adverse cardiovascular and cerebrovascular events), mortality, repeat revascularization, myocardial infarction and stroke in patients suffering from unprotected LMCA and undergoing PCI or CABG surgery. Moreover, a number of major RCTs done on this topic have reported outcomes after updated follow-up periods; hence it is necessary to do a *meta*-analysis taking these studies into account for updated data. Finally, our study aims to provide outcomes for different follow-up lengths including follow-ups for adverse outcomes after 10 years, which has not been provided by previous *meta*-analyses done on this topic.

## 2 Methodology

This *meta*-analysis is reported in concordance with the Preferred Reporting Items for Systematic Reviews and Meta-Analyses (PRISMA) guidelines. This *meta*-analysis only included data from previously published studies, therefore ethical approval was deemed unnecessary.

### 2.1 Search strategy

An electronic search of the MEDLINE, TRIP, and Cochrane Central databases was conducted from their inception to 25 April 2021, without any language restrictions, using a search string containing, but not limited to the terms “left main disease”, “coronary stent” and “bypass surgery”.

No time or language restrictions were used. Moreover, the reference lists of relevant articles were also searched for any other eligible studies. Articles were first shortlisted based on abstracts after which full literature was reviewed to select studies. Bibliographies of the relevant review articles were also queried. In addition to this, grey and white literature was also searched. Articles retrieved from the systematic search were exported to the EndNote Reference Library (Version x7.5; Clarivate Analytics, Philadelphia, Pennsylvania) software, where duplicates were searched for and removed. The remaining articles were carefully assessed by two independent authors (FAJ and SA). A third investigator (ZA) was then consulted to resolve any disparities with consensus. The process for study selection is summarized in the PRISMA flow chart in Supplemental Fig. 2.

**Fig. 1:**
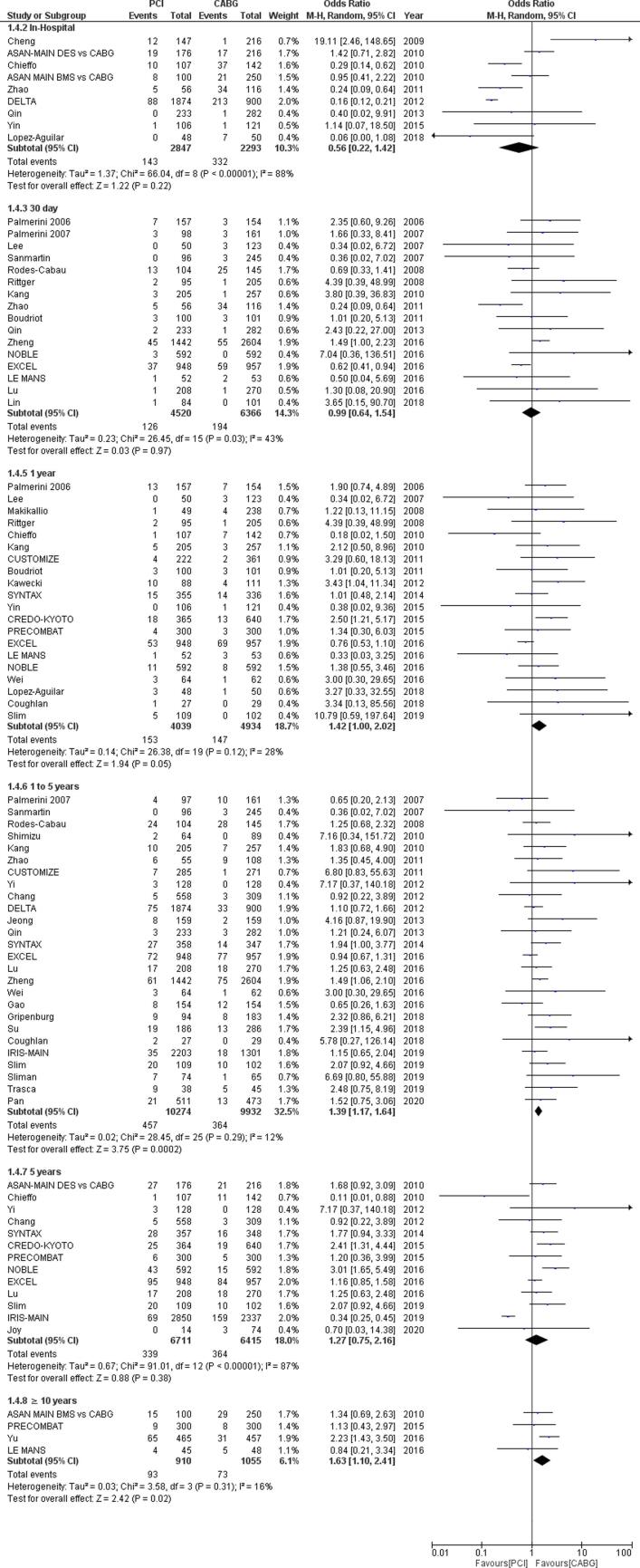
Forest plot for Repeat Revascularization outcome in percutaneous coronary intervention (PCI) versus coronary artery bypass grafting (CABG) for unprotected left main coronary artery disease for varying follow-up lengths.

**Fig. 2:**
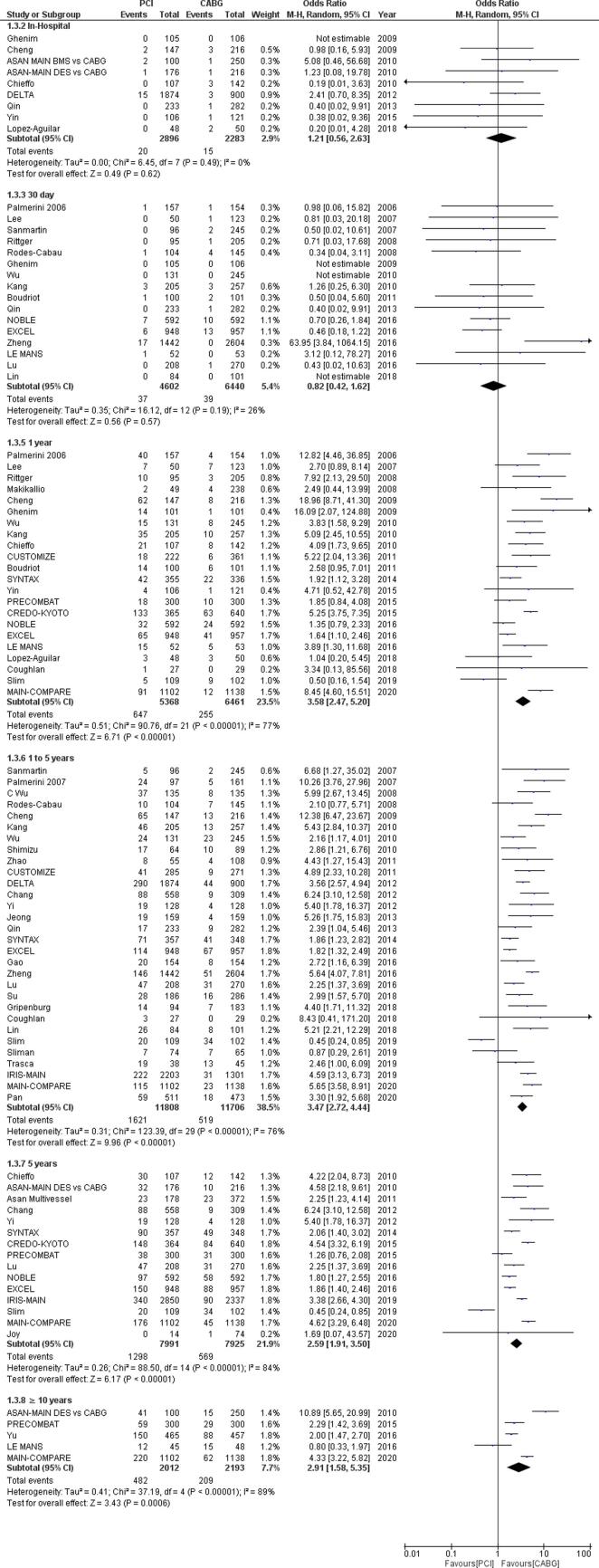
Forest plot for myocardial infarction (MI) outcome in percutaneous coronary intervention (PCI) versus coronary artery bypass grafting (CABG) for unprotected left main coronary artery disease for varying follow-up lengths.

### 2.2 Inclusion and exclusion criteria

The population of interest is patients undergoing a revascularization procedure for unprotected LMD. All RCTs includingopen, single-blind, double-blind, triple-blind, and quadruple blind, and observational studies comparing PCI with drug-eluting or bare-metal stents versus CABG for unprotected LMCAD were selected. Patients undergoing intervention for anything other than LMCAD, animal studies, case reports, conference presentations, editorials, expert opinions, and unpublished studies were excluded. Any duplicate studies from the same database having the same follow-up length [5], [6], [7], [8], [9], [10], [11], [12] as well as studies that did not meet the desired quality according to the quality assessment tools mentioned below (results), were also excluded. [13], [14].

### 2.3 Data extraction and analysis

The data from the selected studies were extracted independently by two authors (AA and HF) and verified by a third author (SY).

From the finalized trials, the following outcomes were assessed: MACCE (major adverse cardiac and cardiovascular events), all-cause mortality, repeat revascularization, myocardial infarction, and stroke. Review Manager (v5.4.1, Copenhagen: The Nordic Cochrane Centre, The Cochrane Collaboration, 2020) was used for all statistical analyses. To visually assess the results of pooling forest plots were constructed. The results were presented as odds ratios and 95% confidence intervals. Subgroups were made for different follow-up lengths including in-hospital follow-up, follow-up after 30 days, short-term (1 year) follow-up, intermediate-term (1 to 5 years) follow-up, long-term (5 years) follow-up, and very long-term (≥10 years) follow-up.

## 3 Results

### 3.1 Search results, study, and patients’ characteristics

Our initial search of the databases yielded 17,281 studies, of which 15,125 were removed after screening titles/abstracts. A total of 57 studies including 7 RCTs (all of which were open blinded) and 50 observational studies published between 2006 and 2021 met our inclusion criteria.

Altogether, clinical data of 56,701 patients who underwent coronary intervention for unprotected left main disease is reported, with 30,259 undergoing PCI and 26,442 undergoing CABG. The characteristics of the selected individual studies and the patients’ baseline characteristics are outlined in the tables below. (Table 1 and Supplemental Tables 3 and 4).

**Table 1:** Characteristics of the included studies.

Author	Year	Study design	PCI (n)	CABG (n)	Region	Outcome	FU (y)
Palmerini 2006 [15]	2006	Observational	157	154	Italy	Mortality, Cardiac Mortality, MI, TLR	2
Palmerini 2007 [16]	2007	Observational	98	161	Italy	Mortality, Cardiac Mortality, MI, TLR	1
Lee [17]	2007	Observational	50	123	USA	MACCE, death, myocardial infarction, urgent TVR,cerebrovascular events, VT / VF, requirement for pacemaker, renal failure, vessel perforation, cardiac tamponade, bleeding	1
Sanmartín [18]	2007	Observational	96	245	Spain	Death,Q-waveMI,Cerebrovascularevents, Repeatrevascularization,MACCE	≥ 1
Brener [19]	2008	Observational	97	190	USA	Mortality	3
C Wu [20]	2008	Observational	135	135	USA	Death, Repeat Revascularization	4
LEMANS Trial [21], [22]	2008–2016	RCT	52	53	America	MACCE, Death, MI, Stroke, Major bleeding	10
MAIN-COMPARE [23], [24], [25], [26]	2008–2018	Observational	1102	1138	Korea	MACCE, Death, MI, Stroke, Repeat Revascularization	10
Makkikalio [27]	2008	Observational	49	238	Finland	Death, Stroke, MI, Repeat Revascularization, MACCE	1
Rittger [28]	2008	Observational	95	205	Germany	MACCE, all cause death, cardiac death, cerebrovascular events, TLR	1
Rodes-Cabau [29]	2008	Observational	104	105	Canada	MACCE, all-cause death, MI, Revascularization, Cerebrovascular events, Life threatening arrythmias, new onset atrial fibrillation, acute renal failure, any bleeding, pleural effusion, respiratory distress, pneumonia	2
White [30]	2008	Observational	67	67	USA	MACCE, All cause mortality	1
Cheng [31]	2009	Observational	147	216	Taiwan	MACCE, All cause mortality, TLR, Cardiac Death, Acute Renal Failure, Ventricular Tachycardia	6
Ghenim [32]	2009	Observational	105	106	France	MACCE, Repeat Revascularization	1
ASAN-MAIN (DES) [33]	2010	Observational	176	219	Korea	Death, Repeat revascularization,Composite point of MI, stroke and TVR	5
Chieffo [34]	2010	Observational	107	142	Italy	MACCE, Death, Cardiac Death, MI, TLR, TVR, Cerebovascular events	5
Kang [35]	2010	Observational	205	257	Korea	All cause death, Cardiac death, Myocardial Infarction, TVR, MACCE	3
Shimizu [36]	2010	Observational	64	89	Japan	MACCE,Death, MI, Stroke, Repeat revascularization, Hospitalization costs	≥1
SYNTAX [37], [38], [39], [40]	2010–2014	RCT	357	348	17 countries	MACCE, Death, Cardiac mortality, MI, Repeat revascularization	10
_Wu_[41]	2010	Observational	131	245	China	Death, TVR, MACCE, MI,Stroke	4
Asan Multivessel [42]	2011	Observational	178	372	Korea	Death, Repeat revascularization, Composite point of MI, stroke and TVR	5
Boudriot [43]	2011	RCT	100	101	Germany	Death, MI, TVR,Any major adverse cardiac event	1
CUSTOMIZE [44], [45]	2011	Observational	285	361	Italy	Major adverse cardiac events, All-cause death, Cardiac Death, MI,TVR, TLR	2
PRECOMBAT Study [46], [47], [48]	2011–2015	RCT	300	300	South Korea	MACCE, MI, Stroke, Death,TVR,Cardiac mortality, Repeat revascularization, Stent thrombosis or symptomatic graft occlusion	2
Zhao [49]	2011	Observational	56	116	China	MACCRE, death, cardiac tamponade, acute MI, acute left heart failure, requirement for pacemaker, VT / VF, pleural effusion, postoperative pneumothorax, shock, required dialysis, repeat thoracotomy, bleeding, vascular hematoma, target vessel revascularization, cerebrovascular events, major adverse cardiac events	≥ 2
Chang [50]	2012	Observational	558	309	Korea	MACCE, Death, MI, Repeat Revascularization, Stroke	5
CREDO-KYOTO[51], [52]	2012–2015	Observational	365	640	Japan	Death, MI, Stroke, Cardiac death, Repeat revascularization	5
DELTA [53]	2012	Observational	1874	900	7 countries	Cardiac death, Non cardiac death, MI, TLR, TVR, Cerebrovascular Accident, MACCE	≥ 1
Kawecki [54]	2012	Observational	88	111	Poland	MACCE, Death, Stroke, ACS	≥ 1
_Yi_[55]	2012	Observational	128	128	Korea	MACCE, TVR,MI, Stroke	5
Gao [56]	2013	Observational	154	154	China	All-Cause Mortality, MI, TVR, Stroke	≥ 2
Jeong [57]	2016	Observational	159	159	South Korea	MACCE including death, stroke, acute myocardial infarction and target-vessel revascularization	≥ 4
Qin [58]	2013	Observational	233	282	China	Death, Cardiac mortality, MI, TVR, Stroke, MACCE	≥ 2
Yin [59]	2015	Observational	106	121	China	MACCE, MI, Stroke, Death, Cardiac mortality	1
EXCELTrial [60], [61]	2016	RCT	948	957	All world	Death, Stroke, Cardiac mortality, MI, Repeat revascularization, TVR, Major bleeding	5
Lu [62]	2016	Observational	208	270	Taiwan	MACCE, All Cause Death, Repeat Revascularization, MI, Stroke, Stent Thrombosis	5
NOBLE study [63], [64]	2016	RCT	592	592	Northern Europe	Death,Cardiac mortality, All-cause mortality, MI, TVR, Stroke, Repeat revascularization	5
Wei [65]	2016	Observational	64	62	China	Cardiac death, Stroke, MACCE	≥ 1
_Yu_[66]	2016	Observational	465	457	China	MACCE, MI, Stroke, Death, Repeat revascularization, Cardiac mortality	10
Zheng [67]	2016	Observational	1442	2604	China	All-cause death, Cardiac mortality, MI, Stroke, Repeat revascularization, TVR	3
IRIS-MAIN [68], [69]	2017	Observational	2850	2337	South Korea	MACCE, Death, MI, Stroke, Repeat Revascularization	5
Coughlan [70]	2018	Observational	27	29	Ireland	MACCE, All Cause Mortality, Stroke, MI, Repeat Revascularization	3
Gripenburg [71]	2018	Observational	94	183	Sweden	All-cause death, MI, Cerebrovascular Accident (CVA), Repeat Revascularization and major bleeding leading to hospital admission.	≥ 2
Lin [72]	2018	Observational	84	101	Taiwan	MACCE, All Cause Mortality, Stroke, MI, Repeat Revascularization, New permanent hemodialysis	3.5
Lopez-Aguilar [73]	2018	Observational	48	50	Mexico	MACCE, MI, all-cause death, cardiac death, myocardial infarction, Repeat Revascularization, cerebrovascular accident, reoperation for bleeding	≥ 1
Obeid [74]	2018	RCT	45	25	Switzerland	NACE, MACCE, Moderate GUMBO bleeding	1 month
Ram [75]	2018	Observational	67	185	Israel	Cardiogenic shock, Permanent Pacemaker implantation, new onset atrial fibrillation, sepsis, all cause mortality	3
Su [76]	2018	Observational	186	286	Taiwan	MACCE, MI, all-cause death, TVR	≥3
Milan [77]	2019	Observational	11	84	Netherlands	Death, Repeat Revascularization or Death	40
Slim [78]	2019	Observational	109	102	Tunisia	MACCE, All Cause Mortality, Stroke, MI, Repeat Revascularization	5
Sliman [79]	2019	Observational	74	65	Israel	MI, Stroke, Repeat Revascularization, Death	3
Trasca [80]	2019	Observational	38	45	Romania	Angina Pectoris, Non fatal MI, All Cause Mortality, LVEF, Repeat Revascularization	3
Joy [81]	2020	Observational	14	74	United Kingdom	MACCE, All Cause Mortality, Stroke, MI, TVR	5
Pan [82]	2020	Observational	511	473	China	MACCE, All Cause Death, Cardiac Death, MI, Stroke, TVR	≥ 2
Song [83]	2020	Observational	149	273	South Korea	MACCE, MI, All Cause Death, Stroke, TVR	10
Mohamed [84]	2021	Observational	13,994	8241	United Kingdom	In-hospital& 30 day mortality	1 month
Xun Wang [85]	2021	Observational	161	207	China	MACCE	3

Abbrevations: PCI = percutaneous coronary intervention; CABG = coronary artery bypass grafting; FU = follow-up; RCT = randomized control trial; MI = myocardial infarction; TLR = target lesionrevascularization; TVR = target vessel revascularization; VT = ventricular tachycardia; VF = ventricular fibrillation; ACS = acute coronary syndrome; CVA = cerebrovascular accident;MACCE = Major adverse cardiac and cerbebrovascular events; MACCRE = Major adverse cardiac, cerebrovascular and renal events.

### 3.2 Quality assessment and publication bias

Both the RCTs and observational studies collected for this pooled analysis were of high quality. The Newcastle-Ottawa scale was used to filter observational studies for quality, while the Cochrane risk of bias tool was used to determine the quality of RCTs. There was no evidence of small study bias [p = 0.322 for Egger’s regression test] (supplementary file, Fig. 3).

**Fig. 3:**
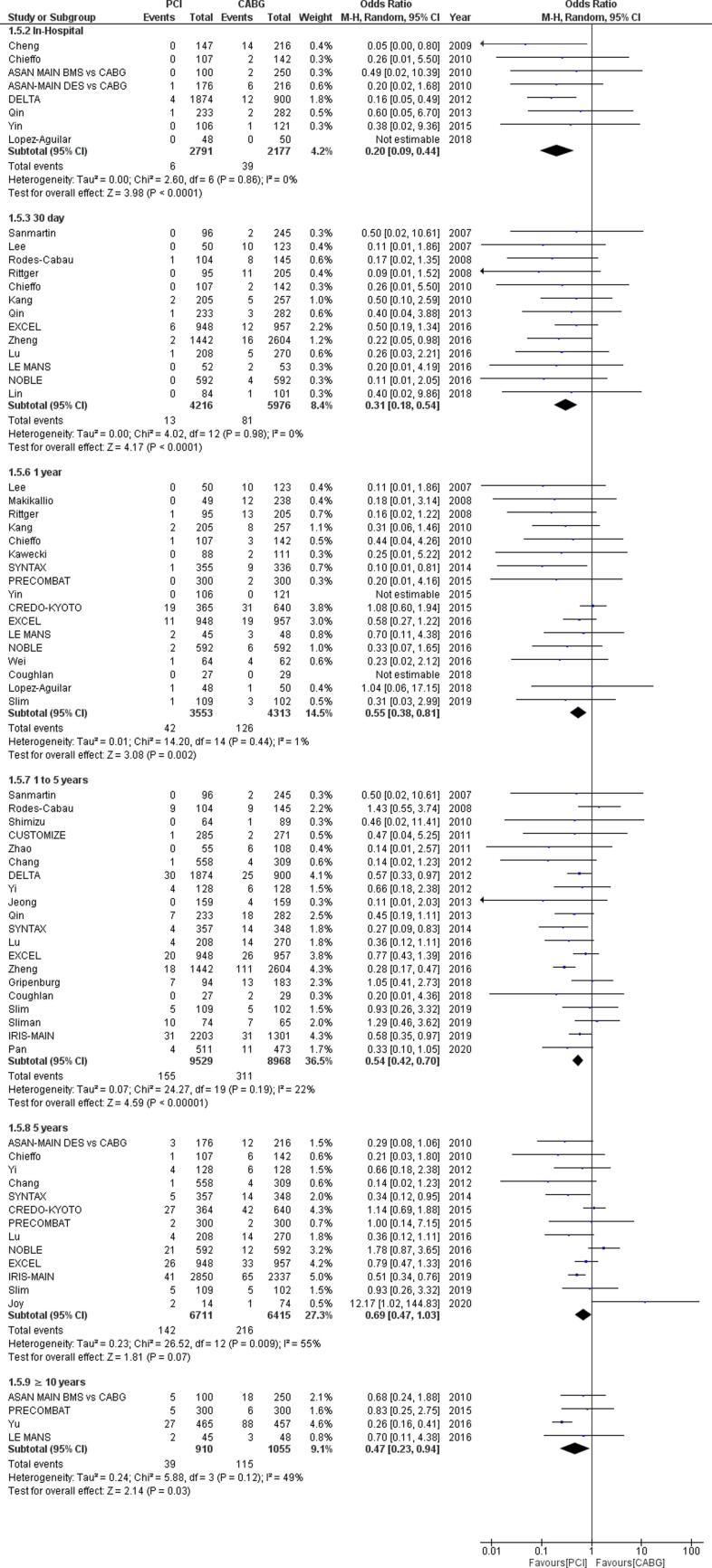
Forest plot for Stroke outcome in percutaneous coronary intervention (PCI) versus coronary artery bypass grafting (CABG) for unprotected left main coronary artery disease for varying follow-up lengths.

## 4 Results of *meta*-analysis

### 4.1 Macce

The definition of MACCE (major adverse cardiac and cerebrovascular events) varies from study to study. For our *meta*-analysis, we only considered studies that reported MACCE as a composite endpoint of all-cause mortality, repeat revascularization, myocardial infarction and stroke. Out of the 57 selected studies, 39 studies (6 RCTs and 33 observational studies) containing data for a total of 44,353 patients, reported outcomes for MACCE. Our pooled analysisin Supplemental Figure 4 shows there was no significant difference in the rate of MACCE post-PCI compared to the rate of MACCE post-CABG during the in-hospital period (OR = 0.64, 95% CI [0.38–1.10]; I ^2^ = 14; p = 0.33), long term follow-up (OR = 1.14, 95% CI [0.90–1.44]; I ^2^ = 81; p = 0.29) or very long term follow-up (OR = 1.10, 95% CI [0.90–1.35]; I ^2^ = 9; p = 0.37). However, PCI was associated with significantly lower rates of MACCE compared to CABG after 30 days of follow-up (OR = 0.41, 95% CI [0.27–0.62]; I ^2^ = 70; p < 0.0001), while PCI had significantly higher rates of MACCE compared to CABG in our short term (OR = 1.23, 95% CI [1.02 – 1.48]; I ^2^ = 42; p = 0.03) and intermediate term follow-up (OR = 1.45, 95% CI [1.21–1.75]; I ^2^ = 73; p < 0.0001).

### 4.2 All-cause mortality

Out of the 57 selected studies, 52 studies (6 RCTs and 46 observational studies), containing data for 118,564 reported data for all-cause mortality. Our pooled analysis in Supplemental Figure 5 shows there was no significant difference in the rates of all-cause mortality following PCI compared to that with CABG during the in-hospital period (OR = 0.67, 95% CI [0.45–1.00]; I ^2^ = 57; p = 0.05), after 30 days (OR = 0.78, 95% CI [0.54–1.12]; I ^2^ = 52; p = 0.17), in our short term follow-up (OR = 0.82, 95% CI [0.64–1.04]; I ^2^ = 39; p = 0.03), intermediate follow-up (OR = 1.08, 95% CI [0.89–1.32]; I ^2^ = 72; p = 0.44), or long term follow-up (OR = 0.89, 95% CI [0.73–1.08]; I ^2^ = 68; p = 0.24). However, in the very long term follow-up, PCI had significantly lower rates of mortality as compared to CABG (OR = 0.77, 95% CI [0.61 – 0.96]; I ^2^ = 46; p = 0.02).

### 4.3 Repeat revascularization

Out of the 57 selected studies, 47 studies (6 RCTs and 41 observational studies) containing data for 71,685 patients reported outcomes for repeat revascularization. Our pooled analysis in Fig. 1 shows there was no significant difference in the rates of repeat revascularization post-PCI compared to the repeat revascularization rates post-CABG during the in-hospital period (OR = 1.21, 95% CI [0.56–2.63]; I ^2^ = 0; p = 0.62) and after 30 days (OR = 0.82, 95% CI [0.42–1.62]; I ^2^ = 26; p = 0.57), however there were significantly higher rates of repeat revascularization for PCI as compared to CABG in the short term follow-up (OR = 3.58, 95% CI [2.47–5.20]; I ^2^ = 77; p < 0.00001), intermediate follow-up (OR = 3.47, 95% CI [2.72–4.44]; I^2^ = 76; p < 0.00001) long term follow-up (OR = 2.58, 95% CI [1.89–3.52]; I ^2^ = 84; p < 0.00001) and very long term follow-up (OR = 2.91, 95% CI [1.58 – 5.35]; I ^2^ = 89; p = 0.0006).

### 4.4 Myocardial infarction

Out of the 57 selected studies, 44 studies (6 RCTs and 38 observational studies) containing data for 60,296 patients reported outcomes for myocardial infarction. Our pooled analysis in Fig. 2 shows that there was no significant difference in the rates of MI post-PCI compared to post-CABG during the in-hospital period (OR = 0.56, 95% CI [0.22–1.42]; I ^2^ = 88; p = 0.22), after 30 days (OR = 0.99, 95% CI [0.64–1.54]; I ^2^ = 43; p = 0.97), in the short-term follow-up (OR = 1.42, 95% CI [1.00–2.02]; I ^2^ = 28; p = 0.05) or long term follow-up (OR = 1.27 95% CI [0.75–2.16]; I ^2^ = 87; p = 0.38) however there were significantly higher rates of MI following PCI as compared to after CABG in the intermediate follow-up (OR = 1.39, 95% CI [1.17–1.64]; I ^2^ = 12; p = 0.0002) and very long term follow-up (OR = 1.63, 95% CI [1.10–2.41]; I^2^ = 16; p = 0.02).

### 4.5 Stroke

Out of the 57 selected studies, 38 studies (5 RCTs and 33 observational studies) containing data for 56,614 patients reported outcomes for stroke. Our pooled analysis in Fig. 3shows that there were significantly lower rates of stroke following PCI as compared to after CABG during the in-hospital period (OR = 0.20, 95% CI [0.09–0.44]; I ^2^ = 0; p < 0.0001), after 30 days (OR = 0.31, 95% CI [0.18–0.54]; I ^2^ = 0; p < 0.0001), in our short term follow-up OR = 0.55, 95% CI [0.38–0.81]; I ^2^ = 1; p = 0.002), intermediate follow-up (OR = 0.54, 95% CI [0.42–0.70] I ^2^ = 22; p < 0.0001) and very long term follow-up. (OR = 0.47, 95% CI [0.23 – 0.94]; I ^2^ = 49; p = 0.03). Although the rates of stroke following PCI were lesser than those following CABG in our long term follow-up as well, this difference was not found to be statistically significant. (OR = 0.69, 95% CI [0.47 – 1.03] I ^2^ = 55; p = 0.07).

## 5 Discussion

Treatment selection for unprotected left main artery disease remains a contentious issue. Several *meta*-analyses, including RCTs with short follow-up periods or observational studies, validated using PCI as a safe and effective alternative over CABG in patients with left artery disease. We accommodated a large number of observational studies and RCTs with a longer follow-up in our study to resolve any discrepancies and overcome deficits in the literature, enhancing generalizability and reliability of our results [86], [87], [88]. To our knowledge, our *meta*-analysis comprising of 57 studies (7 RCTs and 50 observational studies), and 56,701 patients is the largest ever conducted on this topic. It is also the first *meta*-analysis on this topic to provide data for adverse outcomes for a 10 year follow-up. Most previous *meta*-analyses done on this topic only included RCTs, but by considering both RCTs and observational studies, our study provides a more accurate representation of data in clinical settings. Our study also provides updated data from major RCTs (such as the SYNTAX [40], EXCEL [61] and NOBLE [64] trials), that have recently provided data for updated longer follow-up lengths.

Our subgroup analysis suggests that PCI is safer than CABG in terms of stroke in both short-term and long-term follow-up (1–5 years). However, CABG produced significant outcomes in the pooled analysis of MI and repeat revascularization compared to PCI. The results were statistically significant, especially in the long-term follow up (≥1 year). The results proposed that compared to PCI, CABG was associated with higher rates of in-hospital mortality; however, no significant differences were discerned in the rates of all-cause mortality on follow-up duration in patients undergoing PCI or CABG. Major adverse cardiac and cerebrovascular events were detected on long-term follow up (1–5 years) in patients who underwent PCI.

CABG carries a lower risk of mortality in cardiovascular fit individuals. However, the mortality rate associated with CABG increases significantly in older individuals, those requiring repeat vascularization, or those with comorbidities like diabetes and chronic kidney disease [89]. Having said this, previous studies have shown CABG to be safer over PCI in the geriatric population with cardiovascular diseases [90]. This is likely to be due to the fact that these patients have other significant comorbidities that reduce the effectiveness of treatment using stenting.Likewise, data from the BARI (Bypass Angioplasty Revascularization Intervention) trial also supports bypass surgery over PCI for diabetics [91].Thus, it is crucial to provide patients with the best revascularization options after weighing risks and benefits. Our study rendered that there is no difference in the composite outcome of all-cause mortality between the two groups on follow-up. These findings are supported by previous *meta*-analysis including randomized trials [92], [93]. Nevertheless, PCI treatment was associated with improved survival during hospital stay.This can be explained by recent advances in PCI including drug eluting stents and biodegradable stents.This finding is also corroborated by a previous *meta*-analysis that considered 10 randomised trials and concluded that statistics of in-hospital mortality were much higher in patients undergoing CABG with cardiogenic shock compared to PCI [94].However, future trials should update the existing evidence.

PCI was found to be safer than CABG with respect to stroke at almost every length of follow-up. Even though far more episodes of stroke were encountered post-PCI in contrast to post-CABG at 5 years, this difference was not found to be statistically significant. As such, CABG may be used in elderly and diseased individuals, who are at an increased risk of cerebrovascular events. Cerebral embolism secondary to surgical intervention or atrial fibrillation provides the basis for the development of post-CABG stroke [95]. The risk of stroke in the early postoperative days following CABG, in turn, steers the MACCE rates in favor of PCI at 30 days.

Restenosis has been a significant limitation of PCI. Numerous registries showed that repeat revascularization may be necessitated in patients sustaining PCI [96], [97]. Several factors including diabetes mellitus, narrow luminal diameter, complex lesions, and lesions at coronary opening or in the left anterior descending coronary artery are all associated with significantly higher restenosis rates. These elements were identified in several studies included in our synthesis. When compared to PCI, CABG carries a significantly reduced risk of MACCE and MI in patients with unprotected left main coronary artery stenosis at 1–5 years. A possible explanation for this could be that bypassing diseased coronary arteries through graft helps protect the heart against MI, thereby improving survival [98]. The above results are concordant with a previous *meta*-analysis [92].The advantage of CABG over PCI in preventing myocardial infarction was lost at our short and long-term follow-ups, which could be elucidated by losing patients to follow-up. Therefore, further research is warranted to determine whether PCI is a safe and effective alternative to CABG in terms of reducing post-operative MI rates.

Since surgical revascularization with PCI resulted in lower rates of stroke and late mortality but a higher occurrence of MI, LMCAD patients with an increased risk of stroke may opt for PCI over CABG. Despite recent advances in PCI, rates of repeated LMCA revascularization remain high. Due to improved sustainability and durability, CABG remains the appropriate therapeutic intervention for patients who demand long-term survival. Timely identification of perioperative risks and benefits provides better opportunities for patients to choose their treatment options. The 2021 American College of Cardiology / American Heart Association (ACC/ AHA) guidelines for complicated coronary artery lesion currently endorse a multidisciplinary heart team approach (class I indication) in case of ambiguity in choosing between treatment options [99].

Our endeavors were limited in several aspects. Firstly, substantial heterogeneity was recognized in sub-group analysis because of variation in study characteristics, differences in definitions of outcomes, particularly MI and repeat revascularization, and the type of coronary stents used. A random-effect *meta*-analysis was incorporated to address heterogeneity among studies, however heterogeneity remained unchanged. Secondly, few studies did not indicate the type of stent employed. Thirdly, adjunctive medical therapy was not taken into account while comparing PCI and CABG although pharmacological treatment is known to reduce morbidity and mortality. Fourthly, the follow-up period varied drastically across studies hence, clinical end-points were studied at different time intervals (i.e., in-hospital, 30-days, 1-year, 1–5 years, 5 years, and ≥ 10-years). Lastly, clinical health records of individual patients were not accessible to measure the benefits of each revascularization strategy.

## 6 Conclusion

In conclusion, PCI can be considered as a safe alternative over CABG, especially for patients with stroke in the short, intermediate, and very long term follow-ups. CABG however is associated with a lower risk of restenosis in healthy patients. No significant difference was seen in PCI vs CABG in rates of all-cause mortality for most follow-up lengths. However, further research is required for determining whether PCI is a safer alternative over CABG when it comes to preventing episodes of myocardial infarction post-surgery.

## Declaration of Competing Interest

The authors declare that they have no known competing financial interests or personal relationships that could have appeared to influence the work reported in this paper.
