# Supplementary material for: Safety and efficacy of percutaneous coronary intervention versus coronary artery bypass graft in patients with STEMI and unprotected left main stem disease: A systematic review & meta-analysis
Source: Int J Cardiol Heart Vasc. 2022 Apr 25;40:101041. doi: 10.1016/j.ijcha.2022.101041 (PMC9152298; doi:10.1016/j.ijcha.2022.101041)
Supplement: Supplementary Data 1 [file mmc1.docx]

**SUPPLEMENTARY MATERIAL**

**Supplemental Table 1**. Search strategy used in each database searched

| **Database (Articles Retrieved)** | **Search Strategy** |
| --- | --- |
| MEDLINE  (16,385 results) | (("coronary artery disease"[MeSH Terms] OR ("coronary"[All Fields] AND "artery"[All Fields] AND "disease"[All Fields]) OR "coronary artery disease"[All Fields] OR ("Left"[All Fields] AND "main"[All Fields] AND "disease"[All Fields]) OR "left main disease"[All Fields] OR ("Left"[All Fields] AND "main"[All Fields] AND ("constriction, pathologic"[MeSH Terms] OR ("constriction"[All Fields] AND "pathologic"[All Fields]) OR "pathologic constriction"[All Fields] OR "stenosi"[All Fields] OR "stenosis"[All Fields])) OR ("Left"[All Fields] AND "main"[All Fields] AND ("coronary stenosis"[MeSH Terms] OR ("coronary"[All Fields] AND "stenosis"[All Fields]) OR "coronary stenosis"[All Fields] OR ("coronary"[All Fields] AND "artery"[All Fields] AND "stenosis"[All Fields]) OR "coronary artery stenosis"[All Fields])) OR ("LMCA"[All Fields] AND ("constriction, pathologic"[MeSH Terms] OR ("constriction"[All Fields] AND "pathologic"[All Fields]) OR "pathologic constriction"[All Fields] OR "stenosi"[All Fields] OR "stenosis"[All Fields])) OR ("unprotected"[All Fields] AND "Left"[All Fields] AND "main"[All Fields] AND ("constriction, pathologic"[MeSH Terms] OR ("constriction"[All Fields] AND "pathologic"[All Fields]) OR "pathologic constriction"[All Fields] OR "stenosi"[All Fields] OR "stenosis"[All Fields])) OR ("ULM"[All Fields] AND ("constriction, pathologic"[MeSH Terms] OR ("constriction"[All Fields] AND "pathologic"[All Fields]) OR "pathologic constriction"[All Fields] OR "stenosi"[All Fields] OR "stenosis"[All Fields])) OR "ULMCAD"[All Fields] OR ("ULMCA"[All Fields] AND ("disease"[MeSH Terms] OR "disease"[All Fields] OR "diseases"[All Fields] OR "disease s"[All Fields] OR "diseased"[All Fields])) OR ("unprotected"[All Fields] AND ("coronary artery disease"[MeSH Terms] OR ("coronary"[All Fields] AND "artery"[All Fields] AND "disease"[All Fields]) OR "coronary artery disease"[All Fields] OR ("Left"[All Fields] AND "main"[All Fields] AND "disease"[All Fields]) OR "left main disease"[All Fields])) OR ("ULM"[All Fields] AND ("disease"[MeSH Terms] OR "disease"[All Fields] OR "diseases"[All Fields] OR "disease s"[All Fields] OR "diseased"[All Fields])) OR ("LM"[All Fields] AND ("disease"[MeSH Terms] OR "disease"[All Fields] OR "diseases"[All Fields] OR "disease s"[All Fields] OR "diseased"[All Fields]))) AND ("percutaneous coronary intervention"[MeSH Terms] OR ("percutaneous"[All Fields] AND "coronary"[All Fields] AND "intervention"[All Fields]) OR "percutaneous coronary intervention"[All Fields] OR "PCI"[All Fields] OR (("percutaneous"[All Fields] OR "percutaneously"[All Fields] OR "percutanous"[All Fields]) AND ("revascularisation"[All Fields] OR "revascularisations"[All Fields] OR "revascularise"[All Fields] OR "revascularised"[All Fields] OR "revascularising"[All Fields] OR "revascularization"[All Fields] OR "revascularizations"[All Fields] OR "revascularize"[All Fields] OR "revascularized"[All Fields] OR "revascularizes"[All Fields] OR "revascularizing"[All Fields])) OR ("angioplasty, balloon, coronary"[MeSH Terms] OR ("angioplasty"[All Fields] AND "balloon"[All Fields] AND "coronary"[All Fields]) OR "coronary balloon angioplasty"[All Fields] OR ("percutaneous"[All Fields] AND "transluminal"[All Fields] AND "coronary"[All Fields] AND "angioplasty"[All Fields]) OR "percutaneous transluminal coronary angioplasty"[All Fields]) OR ("angioplasty, balloon, coronary"[MeSH Terms] OR ("angioplasty"[All Fields] AND "balloon"[All Fields] AND "coronary"[All Fields]) OR "coronary balloon angioplasty"[All Fields] OR "ptca"[All Fields]) OR (("coronaries"[All Fields] OR "heart"[MeSH Terms] OR "heart"[All Fields] OR "coronary"[All Fields]) AND ("stent s"[All Fields] OR "stentings"[All Fields] OR "stents"[MeSH Terms] OR "stents"[All Fields] OR "stent"[All Fields] OR "stented"[All Fields] OR "stenting"[All Fields]))) AND ("coronary artery bypass"[MeSH Terms] OR ("coronary"[All Fields] AND "artery"[All Fields] AND "bypass"[All Fields]) OR "coronary artery bypass"[All Fields] OR ("coronary"[All Fields] AND "artery"[All Fields] AND "bypass"[All Fields] AND "graft"[All Fields]) OR "coronary artery bypass graft"[All Fields] OR "CABG"[All Fields] OR (("surgical procedures, operative"[MeSH Terms] OR ("surgical"[All Fields] AND "procedures"[All Fields] AND "operative"[All Fields]) OR "operative surgical procedures"[All Fields] OR "surgical"[All Fields] OR "surgically"[All Fields] OR "surgicals"[All Fields]) AND ("revascularisation"[All Fields] OR "revascularisations"[All Fields] OR "revascularise"[All Fields] OR "revascularised"[All Fields] OR "revascularising"[All Fields] OR "revascularization"[All Fields] OR "revascularizations"[All Fields] OR "revascularize"[All Fields] OR "revascularized"[All Fields] OR "revascularizes"[All Fields] OR "revascularizing"[All Fields])) OR (("bypass"[All Fields] OR "bypassed"[All Fields] OR "bypasses"[All Fields] OR "bypassing"[All Fields]) AND ("surgery"[MeSH Subheading] OR "surgery"[All Fields] OR "surgical procedures, operative"[MeSH Terms] OR ("surgical"[All Fields] AND "procedures"[All Fields] AND "operative"[All Fields]) OR "operative surgical procedures"[All Fields] OR "general surgery"[MeSH Terms] OR ("general"[All Fields] AND "surgery"[All Fields]) OR "general surgery"[All Fields] OR "surgery s"[All Fields] OR "surgerys"[All Fields] OR "surgeries"[All Fields])))) AND (1000/1/1:2021/4/25[pdat]) |
| TRIP Database  (1,489 results) | (Left main disease OR Left main stenosis OR Left main coronary artery stenosis OR LMCA stenosis OR unprotected left main stenosis or ULM stenosis OR ULMCAD OR ULMCA disease OR unprotected left main disease or ULM disease OR LM disease) AND (Percutaneous Coronary Intervention OR PCI OR Percutaneous Revascularization OR Percutaneous Transluminal Coronary Angioplasty OR PTCA OR coronary stent) AND (Coronary Artery Bypass graft OR CABG OR Surgical revascularization OR Bypass surgery). |
| Cochrane Central  (440 results) | (Left main disease OR Left main stenosis OR Left main coronary artery stenosis OR LMCA stenosis OR unprotected left main stenosis or ULM stenosis OR ULMCAD OR ULMCA disease OR unprotected left main disease or ULM disease OR LM disease) AND (Percutaneous Coronary Intervention OR PCI OR Percutaneous Revascularization OR Percutaneous Transluminal Coronary Angioplasty OR PTCA OR coronary stent) AND (Coronary Artery Bypass graft OR CABG OR Surgical revascularization OR Bypass surgery). |

| **Supplemental Table 2**. Quality Assesment of included Observational Studies using NewCastle-Ottawa Scale | | | | | | | | | |
| --- | --- | --- | --- | --- | --- | --- | --- | --- | --- |
|  | **Selection** |  |  |  | **Comparability** | **Outcome** |  |  |  |
| **Study** | **Representativeness of the exposed cohort** | **Selection of the non-exposed cohort** | **Ascertainment of exposure** | **Demonstration that the current outcome of interest was not present at start of the study** | **Comparability of cohorts on the basis of the design or analysis** | **Assessment of outcome** | **Was follow-up long enough for outcomes to occur** | **Adequacy of follow-up of cohorts** | **Quality Score** |
| Palmerini 2006^15^ | * | * | * |  | ** |  | * | * | 8 |
| Palmerini 2007^16^ | * |  | * | * | * | * |  | * | 6 |
| Lee^17^ | * | * |  |  | * | * | * | * | 6 |
| Sanmartin^18^ | * | * | * | * | ** | * | * | * | 9 |
| Brener^19^ | * | * | * | * | ** | * | * | * | 7 |
| C Wu^20^ |  | * |  | * | ** | * | * |  | 6 |
| MAIN-COMPARE^23-26^ | * | * |  | * | * |  | * |  | 5 |
| Makkikalio^27^ | * | * | * | * | ** | * | * | * | 9 |
| Rittger^28^ |  | * | * | * | * | * |  |  | 5 |
| Rodes-Cabau^29^ | * |  | * | * | * | * | * | * | 7 |
| White^30^ | * | * |  | * | ** | * | * | * | 8 |
| Cheng^31^ |  |  | * | * | ** | * |  | * | 6 |
| Ghenim^32^ | * | * |  | * | ** |  |  | * | 6 |
| ASAN-MAIN (BMS)^33^ | * | * | * |  | ** |  | * | * | 7 |
| ASAN-MAIN (DES)^33^ | * | * | * | * | * | * | * |  | 7 |
| Chieffo^34^ | * | * | * | * | ** | * | * | * | 9 |
| Kang^35^ | * | * | * | * | * | * | * |  | 7 |
| Shimizu^36^ | * | * | * | * | * | * | * | * | 8 |
| Wu^41^ | * | * |  | * | * |  | * | * | 6 |
| Asan Multivessel^42^ | * | * | * | * | ** | * | * | * | 8 |
| CUSTOMIZE^44,45^ | * | * | * | * | ** | * | * | * | 9 |
| Zhao^49^ | * | * |  | * | * | * |  | * | 5 |
| Chang^50^ |  | * | * | * | ** | * | * |  | 7 |
| CREDO-KYOTO^51,52^ | * | * | * | * | * | * | * | * | 8 |
| DELTA^53^ | * | * |  | * | ** | * |  | * | 6 |
| Kawecki^54^ | * | * | * | * | * | * | * | * | 8 |
| Yi^55^ | * | * |  | * | ** |  | * | * | 7 |
| Gao^56^ | * | * |  | * | * | * | * | * | 7 |
| Jeong^57^ | * | * | * |  | * | * | * |  | 6 |
| Qin^58^ |  | * | * | * | * | * | * | * | 7 |
| Yin^59^ | * | * | * | * | * | * | * | * | 8 |
| Lu^62^ | * | * | * |  | ** | * | * | * | 8 |
| Wei^65^ | * | * |  |  | * | * | * | * | 6 |
| Yu^66^ | * | * | * | * | * | * | * | * | 8 |
| Zheng^67^ | * |  | * | * | * | * | * | * | 6 |
| IRIS-MAIN^68,69^ | * | * | * |  | ** | * | * | * | 8 |
| Coughlan^70^ | * | * | * | * | * |  | * | * | 7 |
| Gripenburg^71^ | * | * | * |  | * | * | * | * | 7 |
| Lin^72^ | * |  |  | * | * | * | * | * | 6 |
| Lopez-Aguilar^73^ | * |  |  | * | ** | * | * |  | 6 |
| Ram^75^ | * |  |  | * | ** |  | * |  | 5 |
| Su^76^ | * | * | * | * | * | * | * | * | 8 |
| Milan^77^ | * | * | * | * | ** | * | * | * | 9 |
| Slim^78^ | * | * | * | * | * |  | * |  | 6 |
| Sliman^79^ | * |  | * | * | * |  | * |  | 5 |
| Trasca^80^ |  | * | * | * | * |  | * | * | 4 |
| Joy^81^ | * |  |  | * | * |  | * |  | 5 |
| Pan^82^ |  | * | * | * | * |  | * | * | 5 |
| Song^83^ |  |  |  | * | ** | * | * | * | 7 |
| Mohamed^84^ |  | * | * |  | * | * |  | * | 6 |


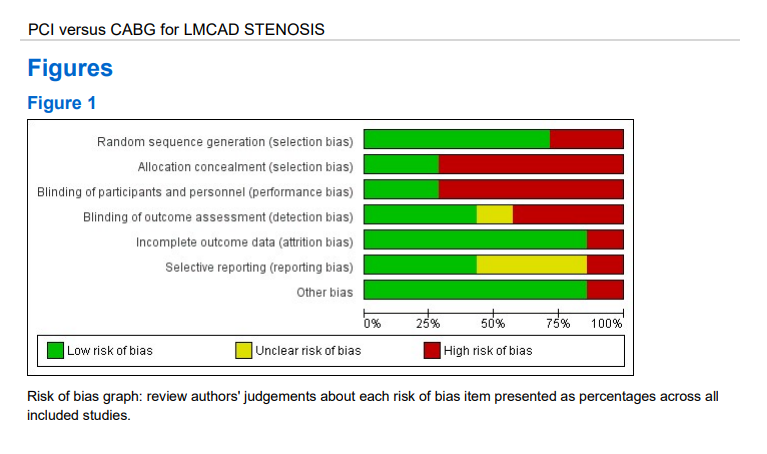


**Supplemental Figure 1a**. Quality Assessment of included Randomized Controlled Trials using Cochrane Risk of Bias Tool


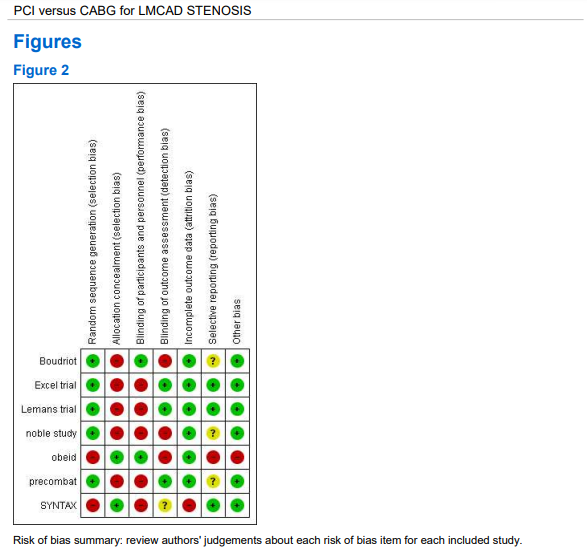


**Supplemental Figure 1b.** Quality Assessment of included Randomized Controlled Trials using Cochrane Risk of Bias Tool

| 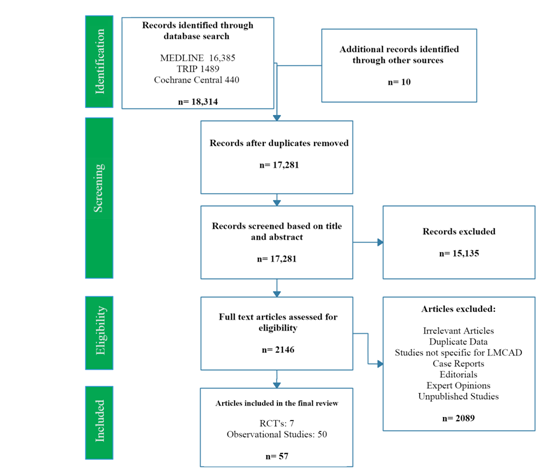  **Supplemental Figure 2**. PRISMA Flow Diagram  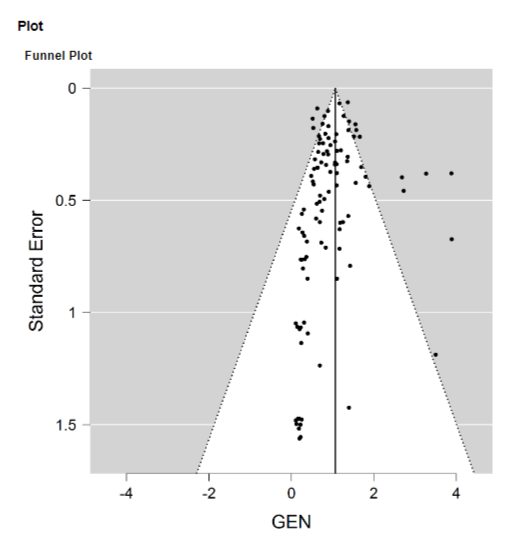  **Supplemental Figure 3.** Funnel Plot to assess publication bias |
| --- |

| **Supplemental Table 3**. Baseline Characteristics of the patients | | | | | | | | | | | | | | | |
| --- | --- | --- | --- | --- | --- | --- | --- | --- | --- | --- | --- | --- | --- | --- | --- |
| **Author** | **Mean Age†** | | **Male (%)**  **PCI / CABG** | **DM (%)**  **PCI / CABG** | **HTN (%)**  **PCI / CABG** |  | **HL (%)**  **PCI / CABG** | **Current or Previous**  **Smokers (%)**  **PCI / CABG** |  | **SYNTAX Score† (Mean)** | |  | **EuroSCORE†**  **(Mean)** | | **Stent**  **Type** |
|  | **PCI CABG** | |  |  |  |  |  |  |  | **PCI CABG** | |  | **PCI CABG** | |  |
|  |  | |  |  |  |  |  |  |  |  | |  |  | |  |
| Palmerini 2006^15^ | 73.0 ± 10.9 | 69.3 ± 9.5 | 70.1 / 76 | 26.1 / 25.3 | 69.4 / 72.7 | | 62.4 / 72.1 | 48.4 / 48.1 | | - | - | | 6 (0-18) | 5 (0-14) | BMS/PES/SES |
| Palmerini 2007^16^ | 81 (75-95) | 78 (75-88) | 54 / 66 | 31 / 26 | 77 / 73 | | 53 / 47 | 39 / 39 | | - | - | | 8 (4–18) | 7 (3–14) | DES |
| Lee^17^ | 72 ± 15 | 70 ± 10 | 50 / 76 | 36 / 31 | 88 / 81 | | 74 / 72 | 12 / 19 | | - | - | | - | - | PES / SES |
| Sanmartín^18^ | 66.0 ± 12.5 | 66.0 ±10.2 | 81 / 87 | 19 / 32 | 44.2 / 60.2 | | 42 / 46 | 38.5 / 45.5 | | - | - | | 4.0 ± 2.5 | 3.9 ± 3.0 | PES / SES |
| Brener^19^ | 68 ± 11 | 68 ± 10 | 72 / 74 | 42 / 25 | 82 / 74 | | - / - | 11 / 57 | | - | - | | 4.6 ± 2.2 | 4.5 ± 2.4 | SES / PES |
| C Wu^20^ | 68.6 ± 11.4 | 68.9 ± 10.8 | 69.6 / 69.6 | 21.5 / 20.7 | - / - | | - / - | - / - | | - | - | | - | - | BMS / DES |
| LEMANS Trial^21,22^ | 60.6 ± 10.5 | 61.3 ± 8.4 | 60 / 73 | 19 / 17 | 75 / 70 | | 65 / 60 | - / - | | 25.2 ± 8.7 | 24.7 ± 6.8 | | 3.3 ± 2.3 | 3.5 ± 2.3 | BMS / DES |
| MAIN-COMPARE^23-26^ | 61.3±11.7 | 62.9±9.4 | 70.7 / 72.9 | 29.7 / 34.7 | 28.6 / 32.6 | | 25.6 / 29.8 | 49.5 /49.4 | | - | - | | - | - | BMS / DES |
| Makkikalio^27^ | 72.9 ± 10 | 70 ± 9 | 59 / 80 | 20 / 17 | 46 / 46 | | - / - | 20 / 18 | | - | - | | 7.7 ± 7.5 | 5.2 ± 4.4 | SES / PES |
| Rittger^28^ | 71.6 ± 9.89 | 68.7 ± 8.99 | 70. 5 / 76.1 | 36.8 / 33.2 | 88.4 / 60.5 | | 56.8 / 94.6 | - / - | | - | - | | 5.07 ± 4.88 | 5.40 ± 6.15 | DES |
| Rodes-Cabau^29^ | 85 ± 3 | 82 ± 2 | 54 / 63 | 27 / 26 | 75 / 72 | | 61 / 82 | 3 / 6 | | - | - | | 9.5 ± 2.6 | 8.4 ± 2.6 | PES/SES/BMS |
| White^30^ | 68.6 ± 13.2 | 72.2 ± 11.9 | 66 / 63 | 34 / 31 | 73 / 78 | | 78 / 75 | 21 / 12 | | - | - | | - | - | SES / PES |
| Cheng^31^ | 68.0 ± 10.4 | 68.0 ± 10.4 | 73.5 / 75.9 | 39.5 / 50.5 | 70.7 / 72.7 | | 68 / 45.8 | 22.4 / 30.6 | | - | - | | 7.4 ± 3.8 | 6.4 ± 3.3 | BMS/SES/PES |
| Ghenim^32^ | 80.7 ± 3.5 | 79.6 ± 3.5 | 36.2 / 28.3 | 30.5 / 23.6 | 73.3 / 65.1 | | 63.8 / 41.5 | 20 / 24.5 | | - | - | | 8 (6-9) | 7 (6-8) | PES |
| ASAN-MAIN (BMS)^33^ | 55.1 ± 10.4 | 60.7 ± 9.1 | 60 / 74.4 | 21 / 32.8 | 23 / 50 | | 34 / 46 | 36 / 27.2 | | - | - | | 3.3 ± 2.1 | 4.4 ± 2.2 | BMS |
| ASAN-MAIN (DES)^33^ | 61.1 ± 11.5 | 62.4 ± 8.1 | 71 / 74 | 29.5 / 37 | 47.2 / 55.3 | | 35.2 / 55.3 | 17.6 / 19.6 | | - | - | | 3.3 ± 2.7 | 4.5 ± 2.6 | SES / PES |
| Chieffo^34^ | 63.6 ± 10.3 | 67.5 ± 9.7 | - / - | 18.7 / 23.2 | 58.8 / 76 | | 70 / 69 | 49.5 / 59.1 | | 28.8 ± 10.4 | 29.4 ± 5.78 | | 4.4 ± 3.6 | 4.3 ± 3.4 | PES / SES |
| Kang^35^ | 64.2 ± 11.5 | 65.7 ± 10 | 70.2 / 73.9 | 37.6 / 43.6 | 63.4 / 67.3 | | 54.6 / 59.5 | 43.4 / 49.4 | | - | - | | 4.2 ± 3.9 | 5.6 ± 3.4 | SES/PES/ZES |
| Shimizu^36^ | 71 ± 7 | 70 ± 9 | 81 / 85 | 35 / 46 | 84 / 79 | | 45 / 58 | 66 / 65 | | - | - | | - | - | SES / PES |
| SYNTAX^37-40^ | 65.4 ± 9.8 | 65.6 ± 10.1 | 72 / 75.6 | 23.8 / 25.6 | 66.9 / 62.4 | | 81 / 75.4 | 17.9 / 24 | | 29.6 ± 13.5 | 30.2 ± 12.7 | | 3.9 ± 2.8 | 3.9 ± 2.9 | DES |
| Wu^41^ | 61.9 ±10.8 | 63.6 ± 9.1 | 76 / 83 | 27 / 29 | 65 / 62 | | 32 / 31 | 39 / 39 | | - | - | | 4.2 ± 2.7 | 4.3 ± 2.4 | DES |
| AsanMultivessel*^42^ | 63 | 63 | 69.4 / 73.2 | 31.6 / 26.9 | 57.1 / 47.9 | | 24.1 / 31.7 | 29.5 / 33.6 | | 17.4 ± 7.8 | 29.9 ± 10.6 | | 3.3 ± 2.4 | 3.9 ± 2.5 | PES / SES |
| Boudriot^43^ | 66 (62-73) | 69 (63-73) | 72 / 77 | 40 / 33 | 82 / 82 | | 68 / 64 | 35 / 28 | | 24 (19-29) | 23 (14.8-28) | | 2.4 (1.5-3.7) | 2.6 (1.7-4.9) | SES |
| CUSTOMIZE^44,45^ | 66.2 ± 11.1 | 65.8 ± 10 | 77.2 / 78.7 | 28.4 / 40.4 | 68.4 / 73.7 | | 56.5 / 52.9 | 44.6 / 44.9 | | 20.1 ± 6.3 | 33.6 ± 13 | | 29.8% > 6 | 5.6 ± 2.5 | DES |
| PRECOMBAT^46-48^ | 61.8 ±10.0 | 62.7 ± 9.5 | 76 / 77 | 34 / 30 | 54.3 / 51.3 | | 42.3 / 40 | 29.7 / 27.7 | | 25 | | | 2.6 ± 1.8 | 2.8 ± 1.9 | SES |
| Zhao^49^ | 51.4-61.5 | 54.8-72 | 73.2 / 71.6 | 100 / 100 | 57.1 / 51.7 | | 44.6 / 42.4 | 50 / 43.5 | | - | - | | - | - | SES / ZES |
| Chang^50^ | 64 ± 10.7 | 65 ± 8.7 | 73.3 / 72.2 | 33 / 39.2 | 59.1 / 55.3 | | 31.7 / 33 | 24.2 / 25.9 | | 25.3 ± 10.2 | 34.5 ± 14.1 | | 3.8 ± 2.5 | 4.2 ± 2.3 | DES |
| CREDO-KYOTO^51,52^ | 71.4 ± 10.1 | 69.4 ± 9.2 | 71 / 77 | 42 / 45 | 86 / 85 | | - / - | 22 / 25 | | 26.5  (21-34) | 30  (22-44) | | - | - | BMS / DES |
| DELTA^53^ | 65.8 ± 11.5 | 66.5 ± 9.8 | 73.9 / 63.6 | 27.7 / 34 | 64 / 67.7 | | 61.8 / 64.7 | 45.8 / 42.7 | | 28.6 ±14.3 | 38.9 ±13.2 | | 4.9 ± 3.6 | 5.1 ± 2.6 | DES |
| Kawecki^54^ | 66.8 ± 10.4 | 65.6 ± 9.1 | 68 / 73 | 30 / 31 | 75 / 72 | | 41 / 41 | 50 / 60.4 | | - | - | | 6.49 ±4.09 | 4.81 ±2.67 | BMS / DES |
| Yi^55^ | 64.7 ± 10.1 | 64.2 ± 8.3 | 78.1 / 72.7 | 32.8 / 31.3 | 59.4 / 62.5 | | - / - | - / - | | - | - | | - | - | DES |
| Gao*^56^ | 61.3 ± 10.4 | 64.5 ± 9.9 | 69.8 / 73.1 | 29.3 / 33.5 | 57.5 / 61 | | 46.5 / 43.9 | 35.6 / 32.4 | | 27.6 + 9.4 | - | | 27.2% > 6 | 22.8% >6 | DES |
| Jeong^57^ | 60.9 ± 9.9 | 60.8 ± 10 | 79.2 / 79.2 | 31.4 / 30.8 | 49.1 / 49.1 | | 1.7 / 1.7 | 20.1 / 20.1 | | 24 ± 8 | 26 ± 7 | | - | - | DES |
| Qin^58^ | 64.9 ± 10.5 | 66.7 ± 8.3 | 84.5 / 87.2 | 24.5 / 27.3 | 56.7 / 69.1 | | 35.2 / 40.1 | 48.1 / 47.2 | | 24.1 ± 10.5 | 34.5 ± 12.0 | | 3.7 ± 2.3 (0-11) | 4.5 ± 2.6 (0-17) | SES/PES/ZES |
| Yin^59^ | 61.67 ± 9.23 | 60.96 ± 6.8 | 67.9 / 56.2 | 21.7 / 21.5 | 66.9 / 65.2 | | 44.34 / 54.55 | 32.1 / 30.5 | | 26.25 ± 4.97 | 32.45 ± 6.06 | | - | - | SES |
| EXCEL Trial^60,61^ | 66.0 ± 9.6 | 65.9 ± 9.5 | 76.2 / 77.5 | 30.2 / 28 | 74.5 / 73.9 | | 71.5 / 69.3 | 24.1 / 20.8 | | 20.6 ± 6.2 | 20.5 ± 6.1 | | - | - | EES |
| Lu^62^ | 70 ± 12 | 69 ± 11 | 84.2 / 85.6 | 47 / 46 | 78 / 83 | | 54 / 50 | 50 / 67 | | - | - | | 7.1 ± 5.1 | 6.4 ± 4.0 | SES / PES /  EES / ZES |
| NOBLE study^63,64^ | 66.2 ± 9.9 | 66.2 ± 9.4 | 80 / 76 | 15 / 15 | 65 / 66 | | 82 / 78 | 19 / 22 | | 22.5 ± 7.5 | 22.4 ± 8 | | 2 (2-4) | 2 (2-4) | BMS / DES |
| Wei[^65^](#_bookmark34) | 73.9 ± 7.4 | 71.0 ± 5.9 | 75 / 79 | 32.8 / 45.2 | 60.9 / 72.6 | | 17.2 / 21 | 39.1 / 39.1 | | 27.3 ± 6.9 | 35.7 ± 6.2 | | 6.8 ± 0.22 | 6.0 ± 0.37 | SES/EES/ZES |
| Yu^66^ | 62 (54-70) | 64 (57-70) | 78.9 / 82.5 | 30.8 / 28.7 | 61.5 / 58.9 | | 49.7 / 34.6 | 49.5 / 44.9 | | - | - | | 5 (3-6) | 5 (3-6) | DES |
| Zheng^67^ | 59.9 ± 10.7 | 62.2 ± 9.1 | 78.6 / 82 | 24.1 / 31 | 54.2 / 64.3 | | 50.1 / 59.1 | 46.5 / 53.6 | | 23.6 ± 6.7 | 33.3 ± 7.8 | | 1.8 ± 1.8 | 2.8 ± 2.1 | DES |
| IRIS-MAIN^68,69^ | 62.7±11.0 | 63.5±9.4 | 74.7 / 76.1 | 32.4 / 37.6 | 57.1 / 57.2 | | 42.1 / 38.9 | 25.2 / 28.5 | | - | - | | - | - | DES |
| Coughlan^70^ | 69 ± 10.4 | 65.1 ± 15.3 | 85 / 82 | 18.5 / 17.2 | - / - | | - / - | - / - | | 33.5 ± 17.6 | 48.4 ± 11.7 | | 4.95 ± 5.8 | 3.11 ± 3.85 | DES |
| Gripenburg^71^ | 72.4 ± 11.6 | 67 ± 8.7 | 79.8 / 85.8 | 16 / 23 | - / - | | - / - | 57.4 / 55.2 | | 30.2 ± 11.1 | 33.3 ± 10.5 | | 2.36  (1.2-5.0) | 1.4  (0.9–2.2) | DES |
| Lin^72^ | 74.2 ± 10 | 72.8 ± 10 | 85.7 / 88.1 | 54.8 / 52.5 | 88.1 / 86.1 | | 52 / 39 | 45 / 70 | | - | - | | 9.9 ± 5.2 | 8.6 ± 3.8 | SES / PES /  EES / ZES |
| Lopez-Aguilar^73^ | 65 ±12 | 66 ±10 | 73 / 86 | 48 / 36 | 52 / 60 | | 46 / 44 | 20 / 32 | | 28 ±12 | 32 ±10 | | (log) 16 ± 21 | (log) 5±6 | DES / BMS |
| Obeid**^74^ | 71.7 ± 10 | | 85.7 | 30 | 78.6 | | 63 | - | | - | - | | - | - |  |
| Ram^75^ | 70 ± 12 | 66 ± 10 | 75 / 81 | 43 / 45 | 82 / 76 | | 82 / 75 | 22 / 25 | | 26 ± 11 | 31 ± 10 | | 3.46 ± 3.17 | 2.64 ± 2.12 | BMS / DES |
| Su^76^ | 77 (66-83) | C-CABG:  70 (60-77) | 80.7 / 83.2 | 45.7 / 47.9 | 79 / 80.1 | | 51.6 / 49.3 | 56.5 / 61.5 | | 30 (24-37) | C-CABG:  37 (30-44) | | - | - | DES / BMS |
|  |  | R-CABG:  66 (59-72) |  |  |  | |  |  | |  | R-CABG: 35 (28-43) | |  |  |  |
| Milan*^77^ | 56.9  (50.4–62.7) | 53.0  (47.7–58.4) | 79.9 / 87.9 | 11.6 / 8.6 | 40.5 / 21.6 | | 27.4 / 22.4 | 58 / 57.8 | | - | - | | - | - | - |
| Slim^78^ | 61.06 ± 11.6 | 61.32 ± 9.1 | 72 / 73 | 58 / 47 | 54 / 56 | | 27 / 34 | 52 / 54 | | 23.3 ± 9.96 | 32.5 ± 8.7 | | 2.21 ± 2.02 | 3.58 ± 9.16 | DES / BMS |
| Sliman^79^ | 86.0±3.5 | 82.7±2.6 | 63 / 57 | 23 / 32 | 92 / 100 | | 85 / 79 | 36 / 15 | | - | - | | - | - | DES |
| Trasca^80^ | 64.32 ± 6.51 | 63.14 ± 6.21 | 36 / 68 | 50 / 38 | 73 / 78 | | 87 / 89 | 75 / 66 | | 27.53 ± 5.80 | 30.06 ± 5.33 | | 2.9 ± 2.4 | 3.4 ± 2.1 | - |
| Joy^81^ | 71 ± 8 | 69 ± 8 | 71 / 92 | 14 / 12 | 29 / 36 | | - / - | 0 / 10 | | 25 ± 8 | 24 ± 9 | | - | - | DES |
| Pan^82^ | 61.02 ± 11.03 | 63 ± 9.09 | 82.2 / 79.7 | 28.4 /26 | 53.4 / 60 | | 15.1 / 20.3 | 45.8 / 40.6 | | - | - | | - | - | DES |
| Song^83^ | 62.4 ± 10.0 | 64.0 ± 8.8 | 36 / 68 | 50 / 38 | 73 / 78 | | 87 / 89 | 75 / 66 | | 24.2 ± 8.5 | 27.2 ± 8.6 | | - | - | DES: 80.5% |
| Mohamed^84^ | 72 (63-79) | 69 (62-75) | 74.4 / 83.3 | 30.3 / 31.6 | 65.6 / 74.7 | | 57.6 / - | 60 / 63.5 | | - | - | | 1.0  (0.5-2.6) | 3.3  (1.9-6.4) | DES: 90.3% |
| Xun Wang^85^ | 67.9 ± 7.9 | 67.6 ± 8.2 | 75.2 / 76.8 | 35.4 / 42 | 85.7 / 82.1 | | 57.8 / 53.1 | 36.6 / 34.8 | | - | - | | - | - | DES |
| * Baseline characteristics of patients in the whole cohort. However, only the left main cohort was analysed  ** Baseline characteristics for the entire left main cohort (PCI + CABG)  † Data provided as mean ± standard deviation or median (IQR)  - : Data not provided  Abbrevations: PCI =percutaneous coronary intervention; CABG = coronary artery bypass grafting; DM = diabetes mellitus; HTN = hypertension; HL = hyperlipidemia / hypercholesterolemia; SYNTAX = Synergy  between Percutaneous Coronary Intervention with TAXUS and Cardiac Surgery; EuroSCORE = European System for Cardiac Operative Risk Evaluation; LEMANS = Left Main Coronary Artery Stenting;  MAIN-COMPARE = Revascularization for Unprotected Left Main Coronary Artery Stenosis: Comparison of Percutaneous Coronary Angioplasty Versus Surgical Revascularization; ASAN-MAIN =ASAN Medical  Center-Left MAIN Revascularization; CUSTOMIZE= The Appraise a Customized Strategy for Left Main Revascularization Registry; PRECOMBAT = Premier of Randomized Comparison of Bypass Surgery Versus  Angioplasty Using Sirolimus-Eluting Stent in Patients With Left Main Coronary Artery Disease ; CREDO-KYOTO = Coronary Revascularization Demonstrating Outcome Study in Kyoto; DELTA = Drug Eluting Stent  for Left Main Coronary Artery; EXCEL=Evaluation of XIENCE versus Coronary Artery Bypass Surgery for Effectiveness of Left Main Revascularization; NOBLE=Nordic-Baltic-British Left Main Revascularization;  IRIS-MAIN =Interventional Research Incorporation Society-Left Main Revascularization registry; BMS= bare-metal stent; DES = drug-eluting stent; SES = sirolimus-eluting stent; PES =paclitaxel-eluting stent;  EES = everolimus-eluting stent; ZES = zotarolimus-eluting stent | | | | | | | | | | | | | | | |

| **Supplemental Table 4.** Pooled patient characteristics for gender, Diabetes Mellitus,Hypertension, Hyperlipidemia and Smokers | | |
| --- | --- | --- |
| **Characteristic [n/sample size, (%)]** | **PCI** | **CABG** |
| Total | 30,259 | 26,442 |
| Male | 22,122 / 29,666 (74.6) | 20,391 /25,665 (79.5) |
| Diabetes Mellitus | 9,152 / 29,871 (30.6) | 8,496 / 25,807 (32.9) |
| Hypertension | 19,078 / 29,615 (64.4) | 17,543 / 26,095 (67.2) |
| Hyperlipidemia | 15,637 / 28,962 (54.0) | 8,356 / 15,949 (52.4) |
| Current / Previous Smokers | 13,703 / 29,434 (47.0) | 11,650 / 25,257 (46.1) |


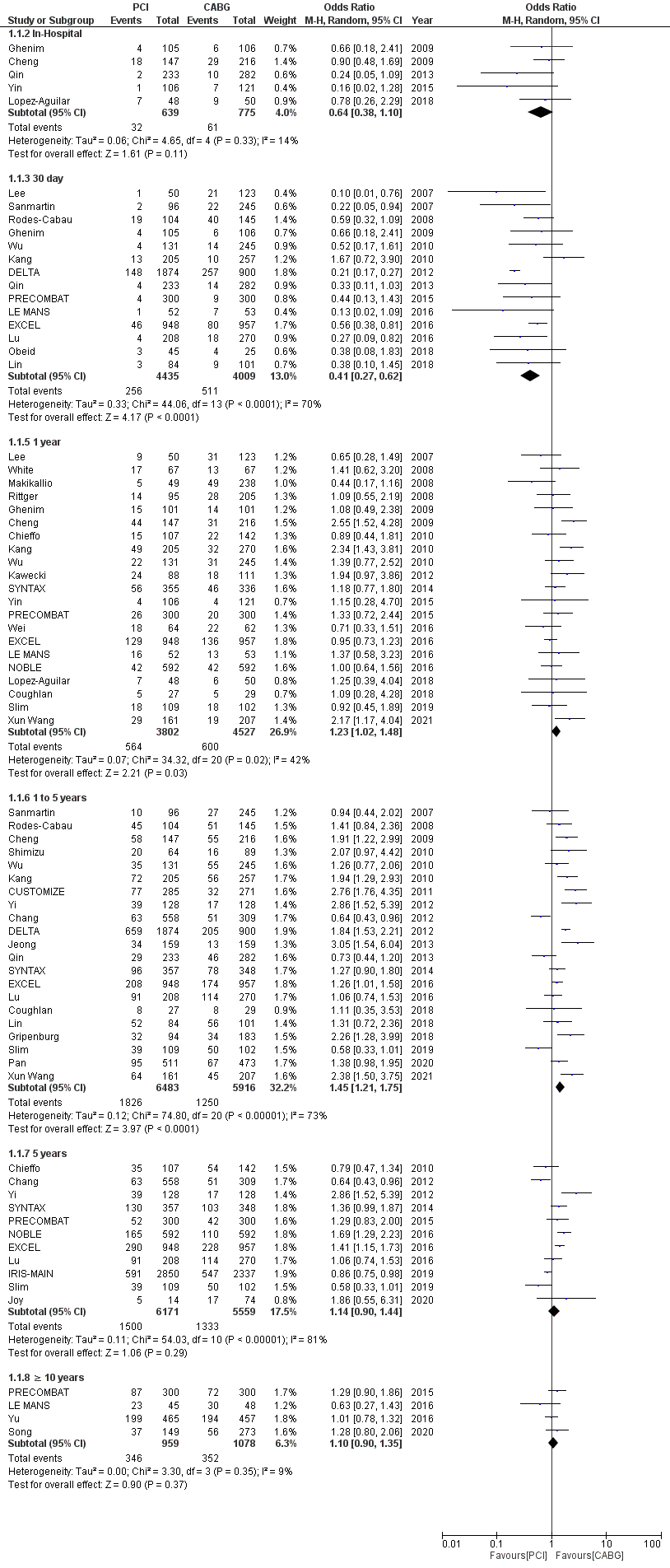


**Supplemental Figure 4**. Forest plot of Major adverse cardiovascular and cerebrovascular events (MACCE) outcome in percutaneous coronary intervention (PCI) versus coronary artery bypass grafting (CABG) for unprotected left main coronary artery disease for varying follow-up lengths


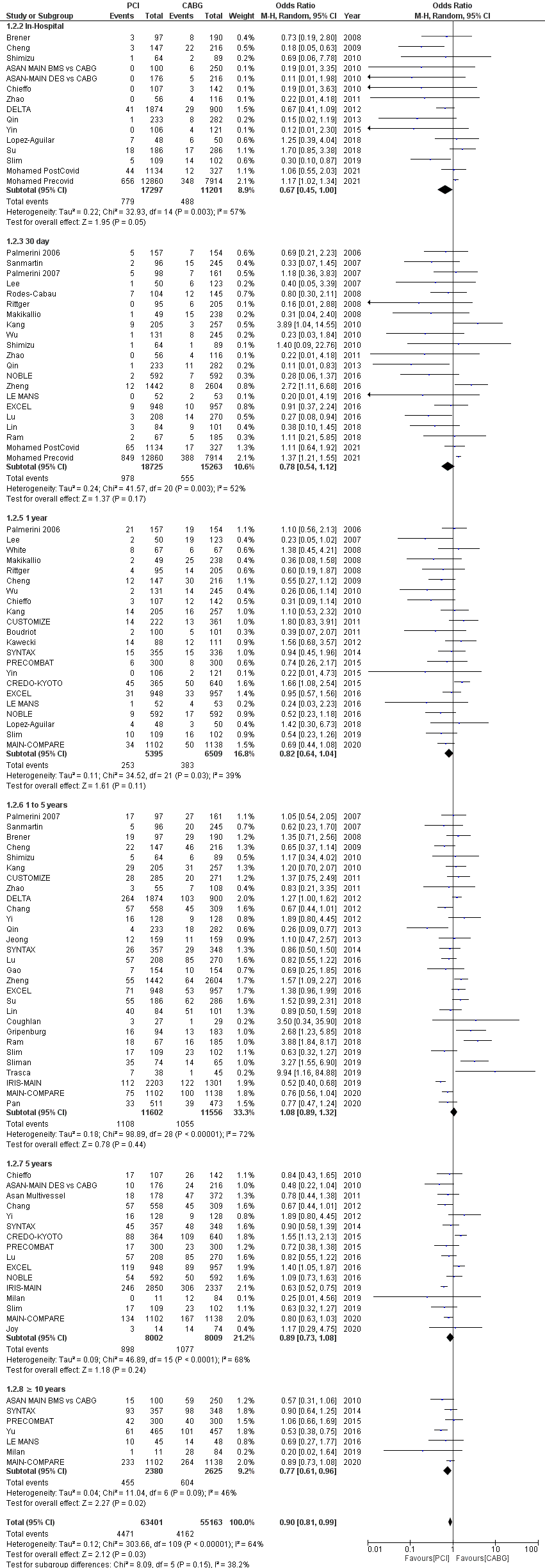


**Supplemental Figure 5.** Forest plot for All-cause Mortality outcome in percutaneous coronary intervention (PCI) versus coronary artery bypass grafting (CABG) for unprotected left main coronary artery disease for varying follow-up lengths
